# The Effects of Antenatal Interventions on Gestational Weight Gain in Low- and Middle-Income Countries: Protocol for a Systematic Review

**DOI:** 10.2196/48234

**Published:** 2023-11-08

**Authors:** Dongqing Wang, Christine H Nguyen, Wafaie W Fawzi

**Affiliations:** 1 Department of Global and Community Health College of Public Health George Mason University Fairfax, VA United States; 2 Department of Global Health and Population Harvard T.H. Chan School of Public Health Harvard University Boston, MA United States; 3 Department of Nutrition Harvard T.H. Chan School of Public Health Harvard University Boston, MA United States; 4 Department of Epidemiology Harvard T.H. Chan School of Public Health Harvard University Boston, MA United States

**Keywords:** low- and middle-income countries, pregnant women, gestational weight gain, maternal nutrition, interventions, randomized controlled trials, systematic review, protocols, RCT, maternal, pregnant, pregnancy, review methodology, nutrition, gestational, weight, prenatal, LMIC, low income, middle income

## Abstract

**Background:**

Gestational weight gain (GWG) is a crucial determinant of maternal and child outcomes yet remains an underused target for antenatal interventions in low- and middle-income countries (LMICs).

**Objective:**

This systematic review aims to identify and summarize educational, behavioral, nutritional, and medical interventions on GWG from randomized controlled trials conducted in LMICs.

**Methods:**

Randomized controlled trials that documented the effects of antenatal interventions on GWG in LMICs will be included. The interventions of interest will be educational, behavioral, nutritional, or medical. A systematic literature search will be conducted using PubMed, Embase, Web of Science, CINAHL (Cumulative Index to Nursing and Allied Health Literature), and the Cochrane Library from the inception of each database through October 2022 (with an updated search in January 2024). A total of 2 team members will independently perform the screening of studies and data extraction. A narrative synthesis of all the included studies will be provided. The risk of bias will be assessed using the Cochrane Risk of Bias tool. The certainty of the evidence for each homogeneous group of interventions will be assessed using the GRADE (Grading of Recommendation, Assessment, Development, and Evaluation) approach. A narrative synthesis of the included studies will be conducted to summarize mean differences (with 95% CIs) for continuous outcomes and risk ratios, rate ratios, hazard ratios, or odds ratios (with 95% CIs) for dichotomous or categorical outcomes. Available information on the costs of interventions will also be summarized to facilitate the adoption and scale-up of effective GWG interventions.

**Results:**

The development of the research questions, search strategy, and search protocol was started on September 20, 2022. The database searches and the importation of the identified records into Covidence were performed on October 7, 2022. As of September 2023, the title and abstract screening was ongoing. The target completion time of this systematic review is April 2024.

**Conclusions:**

Without effective interventions to manage GWG, the potential to improve maternal and child health through optimal GWG remains unrealized in LMICs. This systematic review will inform the design and implementation of antenatal interventions to prevent inadequate and excessive GWG in resource-limited settings.

**Trial Registration:**

PROSPERO (International Prospective Register of Systematic Reviews) CRD42022366354; https://www.crd.york.ac.uk/prospero/display_record.php?RecordID=366354

**International Registered Report Identifier (IRRID):**

PRR1-10.2196/48234

## Introduction

Gestational weight gain (GWG) refers to the gain in body weight during pregnancy. GWG can be measured as total weight gain over the course of pregnancy or the rate of weight gain over a specific period (eg, in a week or in a month). The National Academy of Medicine, formerly the Institute of Medicine (IOM), recommends that women with underweight, normal weight, overweight, and obesity before pregnancy gain, respectively, 12.5 to 18 kg (0.44 to 0.58 kg/week in the second and third trimesters), 11.5 to 16 kg (0.35 to 0.50 kg/week), 7 to 11.5 kg (0.23 to 0.33 kg/week), and 5 to 9 kg (0.17 to 0.27 kg/week) [1]. Weight gain below or above these thresholds is considered inadequate or excessive GWG.

GWG is an important determinant of short- and long-term maternal and child health. On the one hand, pregnant women who gain inadequate gestational weight have elevated risks of small-for-gestational-age births [2], preterm birth [2], low birthweight [3], and infant mortality [4]. On the other hand, pregnant women with excessive GWG experience higher risks of macrosomia [2], large-for-gestational-age births [2], maternal morbidity [5], postpartum weight retention [6], and an increased risk of overweight and obesity among their children [7]. In addition to the critical impacts of GWG on pregnancy outcomes, maternal weight can also be monitored and feasibly modified during pregnancy. Therefore, GWG is a critical indicator for monitoring the overall health of the pregnancy and an important target for antenatal care [1].

Exceptionally high levels of inadequate GWG have been documented in low- and middle-income countries (LMICs). We previously reported that the mean levels of GWG were well below the IOM recommendation in almost all low- and middle-income world regions [8]. The regional level of GWG is particularly low in sub-Saharan Africa and South Asia, both of which meet only around 60% of the IOM recommendation [8]. In the meantime, an emerging burden of excessive GWG has become of concern in LMICs, as several countries in sub-Saharan Africa have over 15% of pregnant women gaining excessive GWG [9]. This concerning trend of excessive GWG may be a consequence of and further compounded by the nutrition transition to Westernized diets, sedentary lifestyles, and the rising prevalence of overweight and obesity [10-12].

Previous interventions on GWG were conducted primarily in high-income countries, often focusing on preventing excessive GWG through lifestyle interventions on dietary intake and physical activity [13-17]. In low- and middle-income settings, the effects of various interventions on inadequate GWG are unclear, and interventions to prevent excessive GWG are scarce. As a result, GWG remains severely underused in antenatal monitoring and intervention in LMICs. An evidence synthesis of interventions for GWG management will fill this critical gap and guide the design and implementation of culturally appropriate and context-relevant interventions on GWG in resource-limited settings.

This systematic review aims to identify and summarize educational, behavioral, nutritional, and medical interventions on GWG from randomized controlled trials (RCTs) in LMICs. This review will provide critical information to inform the design of antenatal interventions or intervention packages aimed at helping pregnant women prevent inadequate and excessive GWG.

## Methods

### Research Question

What are the effects of antenatal interventions (eg, educational, behavioral, nutritional, and medical) on GWG in LMICs?

### Eligibility Criteria

#### Inclusion Criteria

The inclusion criteria were as follows:

1. RCTs, which could be individually randomized, cluster randomized, or have a mixture of individual and cluster randomization.
2. Participants were pregnant at enrollment or enrolled before pregnancy and followed up in pregnancy.
3. Studies conducted in a low-income, lower-middle-income, or upper-middle-income economy defined by the World Bank country classification for the 2023 fiscal year.
4. Interventions provided to women during pregnancy. The intervention could be educational, behavioral, nutritional, or medical. This scope of the interventions is based on the conceptual framework provided by the IOM guidelines regarding the determinants of GWG [1]. Examples of potential interventions include counseling about healthy eating, physical activity, or overall lifestyle; nutrition education on dietary intake; counseling on gestational weight management; anti-infectious regimen; single or multiple micronutrient supplementation; and macronutrient and food supplementation.
5. GWG was included as one of the study outcomes. GWG could be quantified by any metrics, including (but not limited to) total GWG in absolute amount, GWG z-score or adequacy ratio based on references or recommendations, and weekly or monthly rate of GWG. We will include GWG outcomes calculated based on body weights objectively measured by study teams.
6. The intervention could be provided alone or in combination with a cointervention that was similar across study arms.
7. At least 1 arm in the study did not receive the intervention of interest.
8. No restrictions will be placed on the year or sample size of the study.

#### Exclusion Criteria

The exclusion criteria of the studies included:

1. Studies that did not report GWG as 1 of the study outcomes.
2. Studies that used body weight measures based on self-reports or medical record abstraction due to the concern of measurement error.
3. Studies conducted exclusively among women with pre-existing health conditions, such as anemia, human immunodeficiency virus infection, or diabetes. However, studies restricted to women with underweight and women with overweight or obesity will still be included.
4. Observational studies (eg, cohort, case-control, and cross-sectional studies).
5. Interventional studies that did not use individual or cluster randomization.
6. Editorials, commentaries, opinions, or review papers (these will, however, be used to identify relevant original studies).
7. Studies reported in languages other than English.

### Information Source

We will conduct the literature search using PubMed, Embase, Web of Science, CINAHL (Cumulative Index to Nursing and Allied Health Literature), and the Cochrane Library from the inception of each database through October 2022. Our search covers the 3 databases (ie, MEDLINE, Embase, and the Cochrane Library) recommended by the Cochrane Handbook for Systematic Reviews of Interventions [18]. We will also review the references of the previous systematic reviews of a similar topic to locate additional studies.

### Search Strategy

We developed the PubMed search strategy first and then translated the search strategy into the syntax appropriate for the other databases. The search strategies for different databases are provided in Multimedia Appendices 1-5. We examined the sensitivity of the search strategies by confirming that several sentinel papers were identified. The initial search occurred on October 7, 2022, and an updated search will be conducted in January 2024.

### Data Management

We will use EndNote (version 20; Clarivate Analytics) to store the records retrieved from the databases. We will then import the records into Covidence (Veritas Health Innovation), an internet-based program that facilitates the streamlined management of systematic reviews. Duplicate records will be detected and removed first by EndNote and then by Covidence.

### Selection of Studies

A total of 2 team members will independently screen the titles and abstracts of the identified studies based on the eligibility criteria, and 2 team members will then independently review the full texts of the remaining studies to confirm final eligibility. Disagreements between reviewers will be resolved by discussion or by a third reviewer, when necessary, at both the stage of title and abstract screening and the full-text screening. The interrater agreement will be quantified by calculating the raw percentage of agreement and the Cohen κ coefficient. Specific reasons for study exclusions will be documented and summarized using the PRISMA (Preferred Reporting Items for Systematic Reviews and Meta-Analyses) flow diagram [19].

### Data Extraction

We will conduct data extraction using the extraction function in Covidence. A total of 2 reviewers will independently extract the data of the retained studies using a data extraction form that will be pilot-tested on 5 randomly selected studies. Disagreements in the extracted information between reviewers will be resolved by discussion or by a third reviewer. We will extract the following information: study reference, setting and country of the study population, calendar year of intervention, randomization approach, sample size (number of clusters for each group, and number of participants in each group), sample characteristics (eg, age and socioeconomic status), timing and duration of intervention, description of the intervention, control, primary and secondary study outcomes, approach to defining and measuring GWG, main findings (with point estimates and measures of variance), and cost of the intervention (if available). If a publication is missing or unclear on any important study detail, we will contact the corresponding authors of the studies to obtain the information. Multiple reports of a single study will be collated as additional results may be provided in different reports. The data extraction tables are provided in Multimedia Appendices 6 and 7.

### Risk of Bias Assessment

A total of 2 reviewers will independently assess the risk of bias, and any disagreement will be resolved by discussion and by a third reviewer when necessary. We will use version 2 of the Cochrane risk-of-bias tool (RoB 2) [20], which considers the following 5 domains: bias arising from the randomization process, bias due to deviations from intended interventions, bias due to missing outcome data, bias in the measurement of the outcome, and bias in the selection of the reported results. For cluster-randomized trials, we will additionally consider bias from the timing of identification and recruitment of individual participants in relation to the timing of randomization [21]. Each domain will be judged as “low risk of bias,” “high risk of bias,” or “some concerns.” We will consider a study to be of low risk of bias if all domains are judged to have a low risk. We will consider a study to be of high risk of bias if at least 1 domain is judged to have a high risk or ≥3 domains are judged to have some concerns. We will consider a study to have some concerns if 1 or 2 domains are judged to have some concerns, but none of the domains is judged to have a high risk [20]. We will summarize the risk of bias assessment in tabular form and present the judgment for each domain with a justification [20].

### Data Synthesis

A narrative synthesis of all included studies will be presented in the data extraction tables shown in Multimedia Appendices 6 and 7. Effect estimates for continuous outcomes will be expressed as mean differences (with 95% CIs) comparing the intervention group with the control group. Effect estimates for dichotomous and categorical outcomes will be expressed as risk ratios, rate ratios, hazard ratios, or odds ratios (with 95% CIs), comparing the intervention group with the control group. In addition to the narrative synthesis, we will conduct meta-analyses if a consistently defined intervention-outcome effect was reported in at least 3 studies.

### Assessment of Certainty of Evidence

The overall certainty of the evidence for each reasonably homogeneous group of interventions will be assessed using the Grading of Recommendation, Assessment, Development, and Evaluation (GRADE) approach, which considers the risk of bias, publication bias, imprecision, inconsistency, and indirectness [22-27]. The strength of the overall evidence will be judged as high, moderate, low, or very low [22].

### Registration and Reporting

This protocol was registered with PROSPERO (International Prospective Register of Systematic Reviews). The registration ID is CRD42022366354. In the event of protocol amendments, the date of each amendment will be accompanied by a description of the change and its rationale on PROSPERO. We prepared this protocol following the Preferred Reporting Items for Systematic Review and Meta-Analysis Protocols (PRISMA-P) [28]. We will report the systematic review following the Cochrane Handbook for Systematic Reviews of Interventions [18] and the PRISMA guidelines [19].

## Results

We started the development of the research questions, search strategy, and search protocol on September 20, 2022. On October 7, 2022, we performed the searches of all databases based on the search strategies and imported the identified records into Covidence. As of September 2023, the title and abstract screening was ongoing. We identified 33,664 nonduplicate records from all databases. As of September 25, 2023, a total of 8958 (26.6%) records had received 2 votes, and 8351 (24.8%) records had received 1 vote. The target completion time of this systematic review with the complete paper is April 2024.

## Discussion

GWG has critical implications for the short- and long-term health of the mother and the child. Adequate GWG supports the growth and development of the fetus, while both inadequate and excessive GWG lead to adverse outcomes. LMICs have exceptionally high levels of inadequate GWG [8] and an emerging burden of excessive GWG [9]. Previous interventional studies on GWG were predominantly in high-income countries, with a focus on the prevention of excessive weight gain through dietary and physical activity interventions. Due to differences in stature, body composition, dietary practice, food environment, access to obstetric services, and other contextual factors, the effective and appropriate interventions on GWG differ between high-income countries and LMICs. There is, however, a limited understanding of the effective GWG interventions in low- and middle-income contexts. This systematic review will fill this knowledge gap and provide an updated evidence base to guide future interventions on GWG in resource-limited settings.

Food insecurity, inadequate dietary intake, and maternal undernutrition are the main nutritional contributors to inadequate GWG [1]. It is critical to ensure appropriate energy, macronutrient, and micronutrient intake to meet maternal and fetal needs during pregnancy [29]. Several nutritional interventions have been proposed or evaluated to accommodate the increased maternal nutritional needs during pregnancy [30]. Besides nutritional interventions, previous studies in sub-Saharan Africa also suggest alarmingly poor knowledge and practice of gestational weight management among pregnant women [31-33] and antenatal health care providers [34] in LMICs. Therefore, educational and behavioral interventions also hold promise for achieving optimal GWG for pregnant women in resource-limited settings.

In a recent systematic review and meta-analysis, Teede et al [17] evaluated the effects of antenatal lifestyle interventions pertaining to dietary intake and physical activity on GWG based on randomized trials in high-income countries and LMICs from 1990 to 2020. It was reported that, compared to routine antenatal care, overall lifestyle intervention (–1.15 kg; 95% CI –1.40 to –0.91 kg), lifestyle interventions on diet (–2.63 kg; 95% CI –3.87 to –1.40 kg), lifestyle interventions on physical activity (–1.04 kg; 95% CI –1.33 to –0.74 kg), and lifestyle interventions on dietary intake with physical activity (–1.35 kg; 95% CI –1.95 to –0.75 kg), were all associated with lower total GWG [17]. The review focused on lifestyle and behavioral interventions, and few included trials were from LMICs. Our systematic review will complement this work by covering all types of antenatal interventions with an explicit focus on low- and middle-income settings.

This systematic review will have several important strengths. First, we will cover the full spectrum of potential educational, behavioral, nutritional, and medical interventions that have shown promise in previous RCTs. Therefore, this review will contribute useful information to the design of antenatal intervention packages that seek to combine multiple forms of evidence-based interventions. Second, we will consider all metrics of GWG, including total GWG as an absolute value, GWG adequacy ratio based on references and recommendations, and weekly or monthly rate of GWG. As a result, this work will be informative for a nuanced understanding of the potential mechanisms of how different interventions affect the accrual of gestational weight. Finally, we plan to extract available information on the costs of the interventions that will facilitate the adoption and scale-up of effective GWG interventions. Potential limitations of this systematic review include the restriction to studies published in English and the expected heterogeneity of interventions and GWG metrics to be identified from the literature, which may make meta-analyses infeasible.

Without effective interventions to prevent both forms of inappropriate (inadequate and excessive) GWG, the potential to improve maternal and child health through optimal GWG remains unrealized in LMICs. This systematic review will provide a comprehensive and updated body of evidence that will contribute to the design, implementation, and scale-up of practical, effective, and cost-effective antenatal interventions or integrated intervention packages that help pregnant women in LMICs achieve optimal GWG.

## Appendix

Multimedia Appendix 1 PubMed search strategy for interventions on gestational weight gain in low- and middle-income countries.

Multimedia Appendix 2 Embase search strategy for interventions on gestational weight gain in low- and middle-income countries.

Multimedia Appendix 3 Web of Science search strategy for interventions on gestational weight gain in low- and middle-income countries.

Multimedia Appendix 4 CINAHL (Cumulative Index to Nursing and Allied Health Literature) search strategy for interventions on gestational weight gain in low- and middle-income countries.

Multimedia Appendix 5 Cochrane Library search strategy for interventions on gestational weight gain in low- and middle-income countries.

Multimedia Appendix 6 Extraction of basic study characteristics for the systematic review of antenatal interventions on gestational weight gain in low- and middle-income countries.

Multimedia Appendix 7 Extraction of interventions, outcomes, and key findings for the systematic review of antenatal interventions on gestational weight gain in low- and middle-income countries.

## Abbreviations

CINAHL: Cumulative Index to Nursing and Allied Health Literature
GRADE: Grading of Recommendation, Assessment, Development, and Evaluation
GWG: gestational weight gain
IOM: Institute of Medicine
LMIC: low- and middle-income country
PRISMA: Preferred Reporting Items for Systematic Reviews and Meta-Analyses
PRISMA-P: Preferred Reporting Items for Systematic Review and Meta-Analysis Protocols
PROSPERO: International Prospective Register of Systematic Reviews
RCT: randomized controlled trial
RoB: risk-of-bias

## Data Availability

All data generated and analyzed during this study will be included in the published paper or its supplementary files.
