# Supplementary material for: The Effects of Antenatal Interventions on Gestational Weight Gain in Low- and Middle-Income Countries: Protocol for a Systematic Review
Source: JMIR Res Protoc. 2023 Nov 8;12:e48234. doi: 10.2196/48234 (PMC10666019; doi:10.2196/48234)
Supplement: Multimedia Appendix 1 [file resprot_v12i1e48234_app1.docx]

PubMed search strategy for interventions on gestational weight gain in low- and middle-income countries.

| No. | Concept | Search terms | Number of records  (As of October 7, 2022) |
| --- | --- | --- | --- |
| #1 | Trials | “Clinical Trials as Topic”[Mesh] OR “Randomized Controlled Trial”[pt] OR Clinical Trial[pt] OR “Controlled Clinical Trial”[pt] OR “randomized controlled trials as topic” [MeSH] OR “controlled trial*”[tiab] OR intervention*[tiab] OR “random allocation”[MeSH] OR random*[tiab] OR trial*[tiab] OR “Clinical Trial Protocols as Topic”[Mesh] OR “Clinical Trial Protocol”[pt] OR “Clinical Study”[pt] OR “Clinical Studies as Topic”[Mesh] OR “Therapeutic Uses”[Mesh] OR “therapeutic use”[Subheading] | 8,169,996 |
| #2 | Pregnancy | “Pregnancy”[Mesh] OR Pregnanc*[tiab] OR Pregnant[tiab] OR prenatal[tiab] OR gestation*[tiab] or antenatal[tiab] OR “Pregnant Women”[Mesh] OR gravid[tiab] OR obstetric[tiab] OR antepartum[tiab] OR parity[tiab] OR para[tiab] OR childbearing[tiab] | 1,261,904 |
| #3 | Weight / weight gain | “Body Weight”[Mesh] OR Weight[tiab] OR “Body Mass Index”[Mesh] OR “Body Mass Index”[tiab] OR obesity[Mesh] OR obesity[tiab] OR obese[tiab] OR “Thinness”[Mesh] OR underweight[tiab] OR “Malnutrition”[Mesh] OR undernutrition[tiab] OR “Weight Gain”[Mesh] OR “Gestational Weight Gain”[Mesh] OR “Body Weight Changes”[Mesh] OR “Body-Weight Trajectory”[Mesh] OR “Overweight”[Mesh] OR overweight[tiab] OR “normal-weight”[tiab] OR “Anthropometry”[Mesh] OR anthropometr*[tiab] OR “Ideal Body Weight”[Mesh] | 1,758,532 |
| #4 | Low- and middle-income countries | “Developing Countries”[MeSH] OR “developing countr*”[tiab] OR “developing nation*”[tiab] OR “less developed countr*”[tiab] OR “less developed nation*”[tiab] OR “third world nation*”[tiab] OR “third world countr*”[tiab] OR “under developed nation*”[tiab] OR “underdeveloped nation*”[tiab] OR “under developed countr*”[tiab] OR “underdeveloped countr*”[tiab] OR “middle income countr*”[tiab] OR “middle-income countr*”[tiab] OR “middle income nation*”[tiab] OR “middle-income nation*”[tiab] OR “low income countr*”[tiab] OR “low-income countr*”[tiab] OR “low income nation*”[tiab] OR “low-income nation*”[tiab] OR “poor countr*”[tiab] OR “poor nation*”[tiab] OR lmic[tiab] OR lmics[tiab] OR “Africa”[MeSH] OR “Asia”[MeSH] OR “South America”[MeSH] OR “Latin America”[MeSH] OR “Central America”[MeSH] OR africa[tiab] OR asia[tiab] OR “south america*”[tiab] OR “latin america*”[tiab] OR “central america*”[tiab] OR Afghanistan*[tiab] OR Albania*[tiab] OR Algeria*[tiab] OR Samoa*[tiab] OR Angola*[tiab] OR Armenia*[tiab] OR Azerbaijan*[tiab] OR Bangladesh*[tiab] OR Bengali[tiab] OR Belarus*[tiab] OR Belize[tiab] OR Benin[tiab] OR Bhutan*[tiab] OR Bolivia*[tiab] OR Bosnia*[tiab] OR Herzegovina*[tiab] OR Botswana*[tiab] OR Brazil*[tiab] OR Bulgaria*[tiab] OR “Burkina Faso”[tiab] OR Burkinabe[tiab] OR Burundi*[tiab] OR “Cabo Verd*”[tiab] OR “Cape Verd*”[tiab] OR Cambodia*[tiab] OR Cameroon*[tiab] OR “Central African*”[tiab] OR Chad*[tiab] OR China[tiab] OR Chinese[tiab] OR Colombia*[tiab] OR Comoros[tiab] OR Congo[tiab] OR “Costa Rica*”[tiab] OR “Cote d'Ivoire”[tiab] OR “Ivory Coast”[tiab] OR Cuba[tiab] OR Cuban[tiab] OR Djibouti[tiab] OR Dominica*[tiab] OR Ecuador[tiab] OR Egypt*[tiab] OR “El Salvador*”[tiab] OR Eritrea*[tiab] OR Ethiopia*[tiab] OR Fiji*[tiab] OR Gabon*[tiab] OR Gambia*[tiab] OR Georgia*[tiab] OR Ghana*[tiab] OR Grenada*[tiab] OR Guatemala*[tiab] OR Guinea*[tiab] OR Guyan*[tiab] OR Haiti*[tiab] OR Hondura*[tiab] OR India[tiab] OR Indian*[tiab] OR Indonesia*[tiab] OR Iran*[tiab] OR Iraq*[tiab] OR Jamaica*[tiab] OR Jordan*[tiab] OR Kazakh*[tiab] OR Kenya*[tiab] OR Kiribati[tiab] OR “People's Republic of Korea”[tiab] OR “North Korea”[tiab] OR Kosovo[tiab] OR Kosovar*[tiab] OR Kyrgyz*[tiab] OR Lao[tiab] OR Laos[tiab] OR Laotian*[tiab] OR Lebanon[tiab] OR Lebanes*[tiab] OR Lesotho[tiab] OR Liberia*[tiab] OR Libya*[tiab] OR Macedonia*[tiab] OR Madagascar*[tiab] OR Malawi*[tiab] OR Malaysia*[tiab] OR Maldives[tiab] OR Mali[tiab] OR “Marshall Island*”[tiab] OR Mexico[MeSH] OR Mexico[tiab] OR Mexican*[tiab] OR Micronesia*[tiab] OR Moldova*[tiab] OR Mongolia*[tiab] OR Montenegr*[tiab] OR Morocc*[tiab] OR Mozambique[tiab] OR Myanmar[tiab] OR Burmese*[tiab] OR Burma[tiab] OR Namibia*[tiab] OR Nepal*[tiab] OR Nicaragua*[tiab] OR Niger*[tiab] OR Pakistan*[tiab] OR Paraguay*[tiab] OR Peru*[tiab] OR Philippin*[tiab] OR Rwanda*[tiab] OR “Sao Tome”[tiab] OR Principe[tiab] OR Senegal*[tiab] OR Serbia*[tiab] OR “Sierra Leone*”[tiab] OR “Solomon Island*”[tiab] OR Somalia*[tiab] OR “South Africa*”[tiab] OR “Sri Lanka”[tiab] OR “St Lucia”[tiab] OR “Saint Lucia”[tiab] OR “St Vincent”[tiab] OR “Saint Vincent”[tiab] OR Grenad*[tiab] OR Sudan*[tiab] OR Suriname*[tiab] OR Swaziland*[tiab] OR Eswatini*[tiab] OR Syria*[tiab] OR Tajik*[tiab] OR Tanzania*[tiab] OR Zanzibar[tiab] OR Thai*[tiab] OR Timor*[tiab] OR Togo*[tiab] OR Tonga*[tiab] OR Tunisia*[tiab] OR Turkey[tiab] OR Turkish[tiab] OR Turkmen*[tiab] OR Tuvalu*[tiab] OR Uganda*[tiab] OR Ukrain*[tiab] OR Uzbeki*[tiab] OR Vanuatu*[tiab] OR Venezuela*[tiab] OR Vietnam*[tiab] OR “Viet nam*”[tiab] OR “West Bank”[tiab] OR Gaza*[tiab] OR Palestin*[tiab] OR Yemen*[tiab] OR Zambia*[tiab] OR Zimbabw*[tiab] OR “Western Sahara”[tiab] OR Argentin*[tiab] OR Russia*[tiab] OR Maurit*[tiab] OR Palau[tiab] | 2,568,379 |
| Total | #1 AND #2 AND #3 AND #4 |  | 9647 |
