# Supplementary material for: The Effects of Antenatal Interventions on Gestational Weight Gain in Low- and Middle-Income Countries: Protocol for a Systematic Review
Source: JMIR Res Protoc. 2023 Nov 8;12:e48234. doi: 10.2196/48234 (PMC10666019; doi:10.2196/48234)
Supplement: Multimedia Appendix 2 [file resprot_v12i1e48234_app2.docx]

Embase search strategy for interventions on gestational weight gain in low- and middle-income countries.

| No. | Concept | Search terms | Number of records (as of October 7, 2022) |
| --- | --- | --- | --- |
| #1 | Trials | ‘clinical trial’/exp OR ‘randomized controlled trial’/exp OR ‘clinical trial (topic)’/exp OR ‘controlled clinical trial’/exp OR ‘randomized controlled trial (topic)’/exp OR ‘controlled trial*’:ab,ti OR intervention*:ab,ti OR ‘randomization’/exp OR random*:ab,ti OR trial*:ab,ti OR ‘clinical trial protocol’/exp OR ‘clinical study’/exp OR ‘drug therapy’/exp | 16,456,010 |
| #2 | Pregnancy | ‘pregnancy’/exp OR Pregnanc*:ab,ti OR Pregnant:ab,ti OR prenatal:ab,ti OR gestation*:ab,ti or antenatal:ab,ti OR ‘pregnant woman’/exp OR gravid:ab,ti OR obstetric:ab,ti OR antepartum:ab,ti OR ‘parity’/exp OR parity:ab,ti OR para:ab,ti OR childbearing:ab,ti | 1,441,115 |
| #3 | Weight / weight gain | ‘body weight’/exp OR Weight:ab,ti OR ‘body mass’/exp OR ‘Body Mass Index’:ab,ti OR ‘obesity’/exp OR obesity:ab,ti OR obese:ab,ti OR ‘underweight’/exp OR underweight:ab,ti OR ‘malnutrition’/exp OR undernutrition:ab,ti OR ‘body weight gain’/exp OR ‘gestational weight gain’/exp OR ‘body weight change’/exp OR ‘weight trajectory (body weight)’/exp OR overweight:ab,ti OR ‘normal-weight’:ab,ti OR ‘anthropometry’/exp OR anthropometr*:ab,ti OR ‘ideal body weight’/exp | 2,628,183 |
| #4 | Low- and middle-income countries | ‘developing country’/exp OR ‘developing countr*’:ab,ti OR ‘developing nation*’:ab,ti OR ‘less developed countr*’:ab,ti OR ‘less developed nation*’:ab,ti OR ‘third world nation*’:ab,ti OR ‘third world countr*’:ab,ti OR ‘under developed nation*’:ab,ti OR ‘underdeveloped nation*’:ab,ti OR ‘under developed countr*’:ab,ti OR ‘underdeveloped countr*’:ab,ti OR ‘middle income countr*’:ab,ti OR ‘middle-income countr*’:ab,ti OR ‘middle income nation*’:ab,ti OR ‘middle-income nation*’:ab,ti OR ‘low income countr*’:ab,ti OR ‘low-income countr*’:ab,ti OR ‘low income nation*’:ab,ti OR ‘low-income nation*’:ab,ti OR ‘poor countr*’:ab,ti OR ‘poor nation*’:ab,ti OR lmic:ab,ti OR lmics:ab,ti OR ‘Africa’/exp OR ‘Asia’/exp OR ‘South America’/exp OR ‘South and Central America’/exp OR ‘Central America’/exp OR ‘Central American’/exp OR africa:ab,ti OR asia:ab,ti OR ‘south america*’:ab,ti OR ‘latin america*’:ab,ti OR ‘central america*’:ab,ti OR Afghanistan*:ab,ti OR Albania*:ab,ti OR Algeria*:ab,ti OR Samoa*:ab,ti OR Angola*:ab,ti OR Armenia*:ab,ti OR Azerbaijan*:ab,ti OR Bangladesh*:ab,ti OR Bengali:ab,ti OR Belarus*:ab,ti OR Belize:ab,ti OR Benin:ab,ti OR Bhutan*:ab,ti OR Bolivia*:ab,ti OR Bosnia*:ab,ti OR Herzegovina*:ab,ti OR Botswana*:ab,ti OR Brazil*:ab,ti OR Bulgaria*:ab,ti OR ‘Burkina Faso’:ab,ti OR Burkinabe:ab,ti OR Burundi*:ab,ti OR ‘Cabo Verd*’:ab,ti OR ‘Cape Verd*’:ab,ti OR Cambodia*:ab,ti OR Cameroon*:ab,ti OR ‘Central African*’:ab,ti OR Chad*:ab,ti OR China:ab,ti OR Chinese:ab,ti OR Colombia*:ab,ti OR Comoros:ab,ti OR Congo:ab,ti OR ‘Costa Rica*’:ab,ti OR ‘Cote d`Ivoire’:ab,ti OR ‘Ivory Coast’:ab,ti OR Cuba:ab,ti OR Cuban:ab,ti OR Djibouti:ab,ti OR Dominica*:ab,ti OR Ecuador:ab,ti OR Egypt*:ab,ti OR ‘El Salvador*’:ab,ti OR Eritrea*:ab,ti OR Ethiopia*:ab,ti OR Fiji*:ab,ti OR Gabon*:ab,ti OR Gambia*:ab,ti OR Georgia*:ab,ti OR Ghana*:ab,ti OR Grenada*:ab,ti OR Guatemala*:ab,ti OR Guinea*:ab,ti OR Guyan*:ab,ti OR Haiti*:ab,ti OR Hondura*:ab,ti OR India:ab,ti OR Indian*:ab,ti OR Indonesia*:ab,ti OR Iran*:ab,ti OR Iraq*:ab,ti OR Jamaica*:ab,ti OR Jordan*:ab,ti OR Kazakh*:ab,ti OR Kenya*:ab,ti OR Kiribati:ab,ti OR ‘People`s Republic of Korea’:ab,ti OR ‘North Korea’:ab,ti OR Kosovo:ab,ti OR Kosovar*:ab,ti OR Kyrgyz*:ab,ti OR Lao:ab,ti OR Laos:ab,ti OR Laotian*:ab,ti OR Lebanon:ab,ti OR Lebanes*:ab,ti OR Lesotho:ab,ti OR Liberia*:ab,ti OR Libya*:ab,ti OR Macedonia*:ab,ti OR Madagascar*:ab,ti OR Malawi*:ab,ti OR Malaysia*:ab,ti OR Maldives:ab,ti OR Mali:ab,ti OR ‘Marshall Island*’:ab,ti OR ‘Mexico’/exp OR Mexico:ab,ti OR Mexican*:ab,ti OR Micronesia*:ab,ti OR Moldova*:ab,ti OR Mongolia*:ab,ti OR Montenegr*:ab,ti OR Morocc*:ab,ti OR Mozambique:ab,ti OR Myanmar:ab,ti OR Burmese*:ab,ti OR Burma:ab,ti OR Namibia*:ab,ti OR Nepal*:ab,ti OR Nicaragua*:ab,ti OR Niger*:ab,ti OR Pakistan*:ab,ti OR Paraguay*:ab,ti OR Peru*:ab,ti OR Philippin*:ab,ti OR Rwanda*:ab,ti OR ‘Sao Tome’:ab,ti OR Principe:ab,ti OR Senegal*:ab,ti OR Serbia*:ab,ti OR ‘Sierra Leone*’:ab,ti OR ‘Solomon Island*’:ab,ti OR Somalia*:ab,ti OR ‘South Africa*’:ab,ti OR ‘Sri Lanka’:ab,ti OR ‘St Lucia’:ab,ti OR ‘Saint Lucia’:ab,ti OR ‘St Vincent’:ab,ti OR ‘Saint Vincent’:ab,ti OR Grenad*:ab,ti OR Sudan*:ab,ti OR Suriname*:ab,ti OR Swaziland*:ab,ti OR Eswatini*:ab,ti OR Syria*:ab,ti OR Tajik*:ab,ti OR Tanzania*:ab,ti OR Zanzibar:ab,ti OR Thai*:ab,ti OR Timor*:ab,ti OR Togo*:ab,ti OR Tonga*:ab,ti OR Tunisia*:ab,ti OR Turkey:ab,ti OR Turkish:ab,ti OR Turkmen*:ab,ti OR Tuvalu*:ab,ti OR Uganda*:ab,ti OR Ukrain*:ab,ti OR Uzbeki*:ab,ti OR Vanuatu*:ab,ti OR Venezuela*:ab,ti OR Vietnam*:ab,ti OR ‘Viet nam*’:ab,ti OR ‘West Bank’:ab,ti OR Gaza*:ab,ti OR Palestin*:ab,ti OR Yemen*:ab,ti OR Zambia*:ab,ti OR Zimbabw*:ab,ti OR ‘Western Sahara’:ab,ti OR Argentin*:ab,ti OR Russia*:ab,ti OR Maurit*:ab,ti OR palau:ab,ti | 3,325,590 |
| Total | #1 AND #2 AND #3 AND #4 |  | 25,006 |
