# Supplementary material for: The Effects of Antenatal Interventions on Gestational Weight Gain in Low- and Middle-Income Countries: Protocol for a Systematic Review
Source: JMIR Res Protoc. 2023 Nov 8;12:e48234. doi: 10.2196/48234 (PMC10666019; doi:10.2196/48234)
Supplement: Multimedia Appendix 4 [file resprot_v12i1e48234_app4.docx]

CINAHL search strategy for interventions on gestational weight gain in low- and middle-income countries.

| No. | Concept | Search terms | Number of records (As of October 7, 2022) |
| --- | --- | --- | --- |
| #1 | Trials | (MH “Randomized Controlled Trials+”) OR (MH “Clinical Trials+”) OR PT “Randomized Controlled Trial” OR PT “Clinical Trial” OR TI controlled trial* OR AB controlled trial* OR TI intervention* OR AB intervention* OR (MH “Random Assignment”) OR TI random* OR AB random* OR TI trial* OR AB trial* | 1,110,437 |
| #2 | Pregnancy | (MH “Pregnancy+”) OR TI Pregnanc* OR AB Pregnanc* OR TI Pregnant OR AB Pregnant OR TI prenatal OR AB prenatal OR TI gestation* OR AB gestation* or TI antenatal OR AB antenatal OR (MH “Expectant Mothers”) OR TI gravid OR AB gravid OR TI obstetric OR AB obstetric OR TI antepartum OR AB antepartum OR (MH “Parity”) OR TI parity OR AB parity OR TI para OR AB para OR TI childbearing OR AB childbearing | 374,589 |
| #3 | Weight / weight gain | (MH “Body Weight+”) OR TI weight OR AB weight OR (MH “Body Mass Index”) OR TI “Body Mass Index” OR AB “Body Mass Index” OR (MH “Obesity+”) OR TI obesity OR AB obesity OR TI obese OR AB obese OR (MH “Thinness”) OR TI thinness OR AB thinness OR (MH “Malnutrition+”) OR (MH “Undernutrition”) OR TI undernutrition OR AB undernutrition OR (MH “Weight Gain+”) OR (MH “Gestational Weight Gain”) OR (MH “Body Weight Changes+”) OR TI overweight AB overweight OR TI “normal-weight” OR AB “normal-weight” OR (MH “Anthropometry+”) OR TI anthropometr* OR AB anthropometr* | 403,186 |
| #4 | Low- and middle-income countries | (MH “Developing Countries”) OR TI developing countr* OR AB developing countr* OR TI developing nation* OR AB developing nation* OR TI less developed countr* OR AB less developed countr* OR TI less developed nation* OR AB less developed nation* OR TI third world nation* OR AB third world nation* OR TI third world countr* OR AB third world countr* OR TI under developed nation* OR AB under developed nation* OR TI underdeveloped nation* OR AB underdeveloped nation* OR TI under developed countr* OR AB under developed countr* OR TI underdeveloped countr* OR AB underdeveloped countr* MH (“Low and Middle Income Countries”) OR TI middle income countr* OR AB middle income countr* OR TI middle income nation* OR AB middle income nation* OR TI low income countr* OR AB low income countr* OR TI low income nation* OR AB low income nation* OR TI poor countr* OR AB poor countr* OR TI poor nation* OR AB poor nation* OR TI lmic OR AB lmic OR TI lmics OR AB lmics OR (MH “Africa+”) OR (MH “Africa South of the Sahara+”) OR (MH “Asia+”) OR (MH “South America+”) OR (MH “Latin America”) OR (MH “Central America+”) OR TI africa* OR AB africa* OR TI asia* OR AB asia* OR TI south america* OR AB south america* OR TI latin america* OR AB latin america* OR TI central america* OR AB central america* OR MH Afghanistan OR TI Afghanistan* OR AB Afghanistan* OR MH Albania OR TI Albania* OR AB Albania* OR MH Algeria OR TI Algeria* OR AB Algeria* OR (MH “Samoa+”) OR TI samoa* OR AB samoa* OR MH Angola OR TI Angola* OR AB Angola* OR MH Armenia OR TI Armenia* OR AB Armenia* OR MH Azerbaijan OR TI Azerbaijan* OR AB Azerbaijan* OR MH Bangladesh OR TI Bangladesh* OR AB Bangladesh* OR TI Bengali OR AB Bengali OR MH Byelarus OR TI Belarus* OR AB Belarus* OR MH Belize OR TI Belize OR AB Belize OR MH Benin OR TI Benin OR AB Benin OR MH Bhutan OR TI Bhutan* OR AB Bhutan* OR MH Bolivia OR TI Bolivia* OR AB Bolivia* OR MH Bosnia-Herzegovina OR TI Bosnia* OR AB Bosnia* OR TI Herzegovina* OR AB Herzegovina* OR MH Botswana OR TI Botswana* OR AB Botswana* OR MH Brazil OR TI Brazil* OR AB Brazil* OR MH Bulgaria OR TI Bulgaria* OR AB Bulgaria* OR MH Burkina Faso OR TI Burkina Faso OR AB Burkina Faso OR MH Burundi OR TI Burundi* OR AB Burundi* OR MH Cape Verde OR TI Cabo Verd* OR AB Cabo Verd* OR TI Cape Verd* OR AB Cape Verd* OR MH Cambodia OR TI Cambodia* OR AB Cambodia* OR MH Cameroon OR TI Cameroon* OR AB Cameroon* OR MH Central African Republic OR TI Central African* OR AB Central African* OR MH Chad OR TI Chad* OR AB Chad* OR (MH “China+”) OR TI china OR AB china OR MH Chinese OR TI chinese OR AB chinese OR MH Colombia OR TI Colombia* OR AB Colombia* OR TI Comoros OR AB Comoros OR MH Congo OR MH Democratic Republic of the Congo OR TI Congo OR AB Congo OR MH Costa Rica OR TI Costa Rica* OR AB Costa Rica* OR MH Cote d'Ivoire OR TI Cote d'Ivoire OR AB Cote d'Ivoire OR TI Ivory Coast OR AB Ivory Coast OR MH Cuba OR TI Cuba OR AB Cuba OR TI cuban OR AB cuban OR MH Djibouti OR TI Djibouti OR AB Djibouti OR MH Dominica OR TI Dominica* OR AB Dominica* OR MH Ecuador OR TI Ecuador OR AB Ecuador OR MH Egypt OR TI Egypt* OR AB Egypt* OR MH El Salvador OR TI El Salvador* OR AB El Salvador* OR MH Eritrea OR TI Eritrea* OR AB Eritrea* OR MH Ethiopia OR TI Ethiopia* OR AB Ethiopia* OR TI Fiji* OR AB Fiji* OR MH Gabon OR TI Gabon* OR AB Gabon* OR MH Gambia OR TI Gambia* OR AB Gambia* OR (MH “Georgia (Republic)”) OR TI Georgia* OR AB Georgia* OR MH Ghana OR TI Ghana* OR AB Ghana* OR TI Grenada* OR AB Grenada* OR MH Guatemala OR TI Guatemala* OR AB Guatemala* OR MH Guinea OR MH Guinea-Bissau OR MH Papua New Guinea OR MH Equatorial Guinea OR TI Guinea* OR AB Guinea* OR MH Guyana OR TI Guyan* OR AB Guyan* OR MH Haiti OR TI Haiti* OR AB Haiti* OR MH Honduras OR TI Hondura* OR AB Hondura* OR MH India OR TI India OR AB India OR TI Indian* OR AB Indian* OR MH Indonesia OR TI Indonesia* OR AB Indonesia* OR MH Iran OR TI Iran* OR AB Iran* OR MH Iraq OR TI Iraq* OR AB Iraq* OR MH Jamaica OR TI Jamaica* OR AB Jamaica* OR MH Jordan OR TI Jordan* OR AB Jordan* OR MH Kazakhstan OR TI Kazakh* OR AB Kazakh* OR MH Kenya OR TI Kenya* OR AB Kenya* OR TI Kiribati OR AB Kiribati OR MH North Korea OR TI People's Republic of Korea OR AB People's Republic of Korea OR TI North Korea OR AB North Korea OR MH Yugoslavia OR TI Kosovo OR AB Kosovo OR TI Kosovar* OR AB Kosovar* OR TI Kyrgyz* OR AB Kyrgyz* OR MH Laos OR TI Lao OR AB Lao OR TI Laos OR AB Laos OR MH Laotians OR TI Laotian* OR AB Laotian* OR MH Lebanon OR TI Lebanon OR AB Lebanon OR TI Lebanes* OR AB Lebanes* OR MH Lesotho OR TI Lesotho OR AB Lesotho OR MH Liberia OR TI Liberia* OR AB Liberia* OR MH Libya OR TI Libya* OR AB Libya* OR (MH “Macedonia (Republic)”) OR TI Macedonia* OR AB Macedonia* OR MH Madagascar OR TI Madagascar* OR AB Madagascar* OR MH Malawi OR TI Malawi* OR AB Malawi* OR MH Malaysia OR TI Malaysia* OR AB Malaysia* OR TI Maldives OR AB Maldives OR MH Mali OR TI Mali OR AB Mali OR TI Marshall Island* OR AB Marshall Island* OR MH Mauritania OR TI Mauritania* OR AB Mauritania* OR TI Maurit* OR AB Maurit* OR MH Mexico OR TI Mexico OR AB Mexico OR TI Mexican* OR AB Mexican* OR (MH “Micronesia+”) OR TI Micronesia* OR AB Micronesia* OR MH Moldova OR TI Moldova* OR AB Moldova* OR MH Mongolia OR TI Mongolia* OR AB Mongolia* OR TI Montenegr* OR AB Montenegr* OR MH Morocco OR TI Morocc* OR AB Morocc* OR MH Mozambique OR TI Mozambique OR AB Mozambique OR MH Myanmar OR TI Myanmar OR AB Myanmar OR TI Burmese* OR AB Burmese* OR TI Burma OR AB Burma OR MH Namibia OR TI Namibia* OR AB Namibia* OR OR MH Nepal OR TI Nepal* OR AB Nepal* OR MH Nicaragua OR TI Nicaragua* OR AB Nicaragua* OR MH Niger OR MH Nigeria OR TI Niger* OR AB Niger* OR MH Pakistan OR TI Pakistan* OR AB Pakistan* OR MH Paraguay OR TI Paraguay* OR AB Paraguay* OR MH Peru OR TI Peru* OR AB Peru* OR MH Philippines OR TI Philippin* OR AB Philippin* OR MH Rwanda OR TI Rwanda* OR AB Rwanda* OR TI Sao Tome OR AB Sao Tome OR TI Principe OR AB Principe OR MH Senegal OR TI Senegal* OR AB Senegal* OR MH Serbia OR TI Serbia* OR AB Serbia* OR MH Sierra Leone OR TI Sierra Leone* OR AB Sierra Leone* OR (MH “Pacific Islands+”) OR TI Solomon Island* OR AB Solomon Island* OR MH Somalia OR TI Somalia* OR AB Somalia* OR MH South Africa OR TI South Africa* OR AB South Africa* OR MH Sri Lanka OR TI Sri Lanka OR AB Sri Lanka OR TI St Lucia OR AB St Lucia OR TI Saint Lucia OR AB Saint Lucia OR TI St Vincent OR AB St Vincent OR TI Saint Vincent OR AB Saint Vincent OR TI Grenad* OR AB Grenad* OR MH Sudan OR TI Sudan* OR AB Sudan* OR MH Suriname OR TI Suriname* OR AB Suriname* OR MH Swaziland OR TI Swaziland* OR AB Swaziland* OR TI Eswatini* OR AB Eswatini* OR MH Syria OR TI Syria* OR AB Syria* OR MH Tajikistan OR TI Tajik* OR AB Tajik* OR MH Tanzania OR TI Tanzania* OR AB Tanzania* TI Zanzibar OR AB Zanzibar OR MH Thailand OR MH Thai OR TI Thai* OR AB Thai* OR MH Timor OR MH East Timor OR TI Timor* OR AB Timor* OR MH Togo OR TI Togo* OR AB Togo* OR TI Tonga* OR AB Tonga* OR MH Tunisia OR TI Tunisia* OR AB Tunisia* OR MH Turkey OR TI Turkey OR AB Turkey OR TI Turkish OR AB Turkish OR TI Turkmen OR AB Turkmen OR TI Tuvalu* OR AB Tuvalu* OR MH Uganda OR TI Uganda* OR AB Uganda* OR MH Ukraine OR TI Ukrain* OR AB Ukrain* OR MH Uzbekistan OR TI Uzbeki* OR AB Uzbeki* OR TI Vanuatu* OR AB Vanuatu* OR MH Venezuela OR TI Venezuela* OR AB Venezuela* OR MH Vietnam OR TI Vietnam* OR AB Vietnam* OR TI Viet nam* OR AB Viet nam* OR TI West Bank OR AB West Bank OR TI Gaza OR AB Gaza OR TI Palestin* OR AB Palestin* OR MH Yemen OR TI Yemen* OR AB Yemen* OR MH Zambia OR TI Zambia* OR AB Zambia* OR MH Zimbabwe OR TI Zimbabw* OR AB Zimbabw* OR TI Western Sahara OR AB Western Sahara OR MH Argentina OR TI Argentin* OR AB Argentin* OR MH Russia OR TI Russia* OR AB Russia* OR TI Palau AB Palau | 799,629 |
| Total | #1 AND #2 AND #3 AND #4 |  | 3000 |
