# Supplementary material for: The Effects of Antenatal Interventions on Gestational Weight Gain in Low- and Middle-Income Countries: Protocol for a Systematic Review
Source: JMIR Res Protoc. 2023 Nov 8;12:e48234. doi: 10.2196/48234 (PMC10666019; doi:10.2196/48234)
Supplement: Multimedia Appendix 5 [file resprot_v12i1e48234_app5.docx]

Cochrane Library search strategy for interventions on gestational weight gain in low- and middle-income countries.

| No. | Concept | Search terms | Number of records (As of October 7, 2022) |
| --- | --- | --- | --- |
| #1 | Trials | #1 MeSH descriptor: [Clinical Trials as Topic] explode all trees  #2 MeSH descriptor: [Randomized Controlled Trials as Topic] explode all trees  #3 (“controlled trial*”):ti,ab,kw (Word variations have been searched)  #4 (intervention*):ti,ab,kw (Word variations have been searched)  #5 MeSH descriptor: [Random Allocation] explode all trees  #6 (random*):ti,ab,kw (Word variations have been searched)  #7 (trial*):ti,ab,kw (Word variations have been searched)  #8 MeSH descriptor: [Clinical Trial Protocols as Topic] explode all trees  #9 MeSH descriptor: [Clinical Studies as Topic] explode all trees  #10 MeSH descriptor: [Therapeutic Uses] explode all trees  #11 {OR #1-#10} | 1,508,519 |
| #2 | Pregnancy | #12 MeSH descriptor: [Pregnancy] explode all trees  #13 (pregnanc*):ti,ab,kw (Word variations have been searched)  #14 (pregnant):ti,ab,kw (Word variations have been searched)  #15 (prenatal):ti,ab,kw (Word variations have been searched)  #16 (gestation*):ti,ab,kw  #17 (antenatal):ti,ab,kw  #18 MeSH descriptor: [Pregnant Women] explode all trees  #19 (gravid):ti,ab,kw  #20 (obstetric):ti,ab,kw  #21 (antepartum):ti,ab,kw  #22 (parity):ti,ab,kw  #23 (para):ti,ab,kw  #24 (childbearing):ti,ab,kw  #25 {OR #12-#24} | 103,919 |
| #3 | Weight / weight gain | #26 MeSH descriptor: [Body Weight] explode all trees  #27 (weight):ti,ab,kw  #28 MeSH descriptor: [Body Mass Index] explode all trees  #29 (“body-mass index”):ti,ab,kw  #30 MeSH descriptor: [Obesity] explode all trees  #31 (obesity):ti,ab,kw  #32 (obese):ti,ab,kw  #33 MeSH descriptor: [Thinness] explode all trees  #34 (underweight):ti,ab,kw  #35 MeSH descriptor: [Malnutrition] explode all trees  #36 (undernutrition):ti,ab,kw  #37 MeSH descriptor: [Weight Gain] explode all trees  #38 MeSH descriptor: [Gestational Weight Gain] explode all trees  #39 MeSH descriptor: [Body Weight Changes] explode all trees  #40 MeSH descriptor: [Body-Weight Trajectory] explode all trees  #41 MeSH descriptor: [Overweight] explode all trees  #42 (overweight):ti,ab,kw  #43 (“normal-weight”):ti,ab,kw  #44 MeSH descriptor: [Anthropometry] explode all trees  #45 (anthropometr*):ti,ab,kw  #46 MeSH descriptor: [Ideal Body Weight] explode all trees  #47 {OR #26-#46} | 184,183 |
| #4 | Low- and middle-income countries | #48 MeSH descriptor: [Developing Countries] explode all trees  #49 (“developing countr*”):ti,ab,kw  #50 (“developing nation*”):ti,ab,kw  #51 (“less developed countr*”):ti,ab,kw  #52 (“less developed nation*”):ti,ab,kw  #53 (“third world nation*”):ti,ab,kw  #54 (“third world countr*”):ti,ab,kw  #55 (“under developed nation*”):ti,ab,kw  #56 (“underdeveloped nation*”):ti,ab,kw  #57 (“under developed countr*”):ti,ab,kw  #58 (“underdeveloped countr*”):ti,ab,kw  #59 (“middle income countr*”):ti,ab,kw  #60 (“middle-income countr*”):ti,ab,kw  #61 (“middle income nation*”):ti,ab,kw  #62 (“middle-income nation*”):ti,ab,kw  #63 (“low income countr*”):ti,ab,kw  #64 (“low-income countr*”):ti,ab,kw  #65 (“low income nation*”):ti,ab,kw  #66 (“low-income nation*”):ti,ab,kw  #67 (“poor countr*”):ti,ab,kw  #68 (“poor nation*”):ti,ab,kw  #69 (lmic):ti,ab,kw  #70 (lmics):ti,ab,kw  #71 MeSH descriptor: [Africa] explode all trees  #72 MeSH descriptor: [Asia] explode all trees  #73 MeSH descriptor: [South America] explode all trees  #74 MeSH descriptor: [Latin America] explode all trees  #75 MeSH descriptor: [Central America] explode all trees  #76 (africa):ti,ab,kw  #77 (asia):ti,ab,kw  #78 (“south america*”):ti,ab,kw  #79 (“central america*”):ti,ab,kw  #80 (“latin america*”):ti,ab,kw  #81 (Afghanistan*):ti,ab,kw  #82 (Albania*):ti,ab,kw  #83 (Algeria*):ti,ab,kw  #84 (Samoa*):ti,ab,kw  #85 (Angola*):ti,ab,kw  #86 (Armenia*):ti,ab,kw  #87 (Azerbaijan*):ti,ab,kw  #88 (Bangladesh*):ti,ab,kw  #89 (Bengali):ti,ab,kw  #90 (Belarus*):ti,ab,kw  #91 (Belize):ti,ab,kw  #92 (Benin):ti,ab,kw  #93 (Bhutan*):ti,ab,kw  #94 (Bolivia*):ti,ab,kw  #95 (Bosnia*):ti,ab,kw  #96 (Herzegovina*):ti,ab,kw  #97 (Botswana*):ti,ab,kw  #98 (Brazil*):ti,ab,kw  #99 (Bulgaria*):ti,ab,kw  #100 (“Burkina Faso”):ti,ab,kw  #101 (Burkinabe):ti,ab,kw  #102 (Burundi*):ti,ab,kw  #103 (“Cape Verd*”):ti,ab,kw  #104 (“Cabo Verd*”):ti,ab,kw  #105 (Cambodia*):ti,ab,kw  #106 (Cameroon*):ti,ab,kw  #107 (“Central African*”):ti,ab,kw  #108 (Chad*):ti,ab,kw  #109 (China):ti,ab,kw  #110 (Chinese):ti,ab,kw  #111 (Colombia*):ti,ab,kw  #112 (Comoros):ti,ab,kw  #113 (Congo):ti,ab,kw  #114 (“Costa Rica*”):ti,ab,kw  #115 (“Cote d'Ivoire”):ti,ab,kw  #116 (“Ivory Coast”):ti,ab,kw  #117 (Cuba):ti,ab,kw  #118 (Cuban):ti,ab,kw  #119 (Djibouti):ti,ab,kw  #120 (Dominica*):ti,ab,kw  #121 (Ecuador):ti,ab,kw  #122 (Egypt*):ti,ab,kw  #123 (“El Salvador*”):ti,ab,kw  #124 (Eritrea*):ti,ab,kw  #125 (Ethiopia*):ti,ab,kw  #126 (Fiji*):ti,ab,kw  #127 (Gabon*):ti,ab,kw  #128 (Gambia*):ti,ab,kw  #129 (Georgia*):ti,ab,kw  #130 (Ghana*):ti,ab,kw  #131 (Grenada*):ti,ab,kw  #132 (Guatemala*):ti,ab,kw  #133 (Guinea*):ti,ab,kw  #134 (Guyan*):ti,ab,kw  #135 (Haiti*):ti,ab,kw  #136 (Hondura*):ti,ab,kw  #137 (India):ti,ab,kw  #138 (Indian*):ti,ab,kw  #139 (Indonesia*):ti,ab,kw  #140 (Iran*):ti,ab,kw  #141 (Iraq*):ti,ab,kw  #142 (Jamaica*):ti,ab,kw  #143 (Jordan*):ti,ab,kw  #144 (Kazakh*):ti,ab,kw  #145 (Kenya*):ti,ab,kw  #146 (Kiribati):ti,ab,kw  #147 (“People's Republic of Korea”):ti,ab,kw  #148 (“North Korea”):ti,ab,kw  #149 (Kosovo):ti,ab,kw  #150 (Kosovar*):ti,ab,kw  #151 (Kyrgyz*):ti,ab,kw  #152 (Lao):ti,ab,kw  #153 (Laos):ti,ab,kw  #154 (Laotian*):ti,ab,kw  #155 (Lebanon):ti,ab,kw  #156 (Lebanes*):ti,ab,kw  #157 (Lesotho):ti,ab,kw  #158 (Liberia*):ti,ab,kw  #159 (Libya*):ti,ab,kw  #160 (Macedonia*):ti,ab,kw  #161 (Madagascar*):ti,ab,kw  #162 (Malawi*):ti,ab,kw  #163 (Malaysia*):ti,ab,kw  #164 (Maldives):ti,ab,kw  #165 (Mali):ti,ab,kw  #166 (“Marshall Island*”):ti,ab,kw  #167 (Mexico):ti,ab,kw  #168 MeSH descriptor: [Mexico] explode all trees  #169 (Mexican*):ti,ab,kw  #170 (Micronesia*):ti,ab,kw  #171 (Moldova*):ti,ab,kw  #172 (Mongolia*):ti,ab,kw  #173 (Montenegr*):ti,ab,kw  #174 (Morocc*):ti,ab,kw  #175 (Mozambique):ti,ab,kw  #176 (Myanmar):ti,ab,kw  #177 (Burmese*):ti,ab,kw  #178 (Burma):ti,ab,kw  #179 (Namibia*):ti,ab,kw  #180 (Nepal*):ti,ab,kw  #181 (Nicaragua*):ti,ab,kw  #182 (Niger*):ti,ab,kw  #183 (Pakistan*):ti,ab,kw  #184 (Paraguay*):ti,ab,kw  #185 (Peru*):ti,ab,kw  #186 (Philippin*):ti,ab,kw  #187 (Rwanda*):ti,ab,kw  #188 (“Sao Tome”):ti,ab,kw  #189 (Principe):ti,ab,kw  #190 (Senegal*):ti,ab,kw  #191 (Serbia*):ti,ab,kw  #192 (“Sierra Leone*”):ti,ab,kw  #193 (“Solomon Island*”):ti,ab,kw  #194 (Somalia*):ti,ab,kw  #195 (“South Africa*”):ti,ab,kw  #196 (“Sri Lanka”):ti,ab,kw  #197 (“St Lucia”):ti,ab,kw  #198 (“Saint Lucia”):ti,ab,kw  #199 (“St Vincent”):ti,ab,kw  #200 (“Saint Vincent”):ti,ab,kw  #201 (Grenad*):ti,ab,kw  #202 (Sudan*):ti,ab,kw  #203 (Suriname*):ti,ab,kw  #204 (Swaziland*):ti,ab,kw  #205 (Eswatini*):ti,ab,kw  #206 (Syria*):ti,ab,kw  #207 (Tajik*):ti,ab,kw  #208 (Tanzania*):ti,ab,kw  #209 (Zanzibar):ti,ab,kw  #210 (Thai*):ti,ab,kw  #211 (Timor*):ti,ab,kw  #212 (Togo*):ti,ab,kw  #213 (Tonga*):ti,ab,kw  #214 (Tunisia*):ti,ab,kw  #215 (Turkey):ti,ab,kw  #216 (Turkish):ti,ab,kw  #217 (Turkmen*):ti,ab,kw  #218 (Tuvalu*):ti,ab,kw  #219 (Uganda*):ti,ab,kw  #220 (Ukrain*):ti,ab,kw  #221 (Uzbeki*):ti,ab,kw  #222 (Vanuatu*):ti,ab,kw  #223 (Venezuela*):ti,ab,kw  #224 (Vietnam*):ti,ab,kw  #225 (“Viet nam*”):ti,ab,kw  #226 (“West Bank”):ti,ab,kw  #227 (Gaza*):ti,ab,kw  #228 (Palestin*):ti,ab,kw  #229 (Yemen*):ti,ab,kw  #230 (Zambia*):ti,ab,kw  #231 (Zimbabw*):ti,ab,kw  #232 (“Western Sahara”):ti,ab,kw  #233 (Argentin*):ti,ab,kw  #234 (Russia*):ti,ab,kw  #235 (Maurit*):ti,ab,kw  #236 (Palau):ti,ab,kw  #237 {OR #48-#236} | 146,139 |
| Total | #1 AND #2 AND #3 AND #4 | #11 AND #25 AND #47 AND #237 | 4021 (3677 trials) |
