# Supplementary material for: The Effects of Antenatal Interventions on Gestational Weight Gain in Low- and Middle-Income Countries: Protocol for a Systematic Review
Source: JMIR Res Protoc. 2023 Nov 8;12:e48234. doi: 10.2196/48234 (PMC10666019; doi:10.2196/48234)
Supplement: Multimedia Appendix 6 [file resprot_v12i1e48234_app6.docx]

Extraction of basic study characteristics for the systematic review of antenatal interventions on gestational weight gain in low- and middle-income countries.

| Study | Setting | Year of study | Study design (individual or cluster randomization) | Sample characteristics (eg, age and socioeconomic status) | Sample size |
| --- | --- | --- | --- | --- | --- |
|  |  |  |  |  |  |
|  |  |  |  |  |  |
|  |  |  |  |  |  |
|  |  |  |  |  |  |
|  |  |  |  |  |  |
|  |  |  |  |  |  |
|  |  |  |  |  |  |
