# Supplementary material for: The Effects of Antenatal Interventions on Gestational Weight Gain in Low- and Middle-Income Countries: Protocol for a Systematic Review
Source: JMIR Res Protoc. 2023 Nov 8;12:e48234. doi: 10.2196/48234 (PMC10666019; doi:10.2196/48234)
Supplement: Multimedia Appendix 7 [file resprot_v12i1e48234_app7.docx]

Extraction of interventions, outcomes, and key findings for the systematic review of antenatal interventions on gestational weight gain in low- and middle-income countries.

| Study | Intervention | Intervention timing and duration | Control | Primary and secondary study outcomes | Definition of GWG and frequency for weight measurements | Main findings | Cost of intervention |
| --- | --- | --- | --- | --- | --- | --- | --- |
|  |  |  |  |  |  |  |  |
|  |  |  |  |  |  |  |  |
|  |  |  |  |  |  |  |  |
|  |  |  |  |  |  |  |  |
|  |  |  |  |  |  |  |  |
|  |  |  |  |  |  |  |  |
|  |  |  |  |  |  |  |  |
